# Targeting of the COX-2/PGE2 axis enhances the antitumor activity of T7 peptide *in vitro* and *in vivo*

**DOI:** 10.1080/10717544.2021.1914776

**Published:** 2021-04-30

**Authors:** Jianrong Yang, Jingtao Zhong, Mi Zhou, Yinghong Zhou, Peng Xiu, Feng Liu, Fuhai Wang, Zelun Li, Yuntian Tang, Yuanyuan Chen, Siyang Yao, Tao Huang, Tianqi Liu, Xiaofeng Dong

**Affiliations:** a Department of Hepatobiliary, Pancreas and Spleen Surgery, The People’s Hospital of Guangxi Zhuang Autonomous Region, Nanning, China; b Department of Hepatobiliary Surgery, Shandong Cancer Hospital and Institute, Shandong First Medical University and Shandong Academy of Medical Sciences, Jinan, China; c Department of Vascular Surgery, Xuanwu Hospital, Capital Medical University, Beijing, China; d School of Medicine and Life Sciences, University of Jinan-Shandong Academy of Medical Sciences, Jinan, China; e Department of General Surgery, Shandong Provincial Qianfoshan Hospital, The First Affiliated Hospital of Shandong First Medical University, Jinan, China

**Keywords:** Tumstatin, T7 peptide, integrin, cyclooxygenase-2, hypoxia

## Abstract

T7 peptide is considered as an antiangiogenic polypeptide. The presents study aimed to further detect the antiangiogenic mechanisms of T7 peptide and determine whether combining T7 peptide and meloxicam (COX-2/PGE2 specific inhibitor) could offer a better therapy to combat hepatocellular carcinoma (HCC). T7 peptide suppressed the proliferation, migration, tube formation, and promoted the apoptosis of endothelial cells under both normoxic and hypoxic conditions via integrin α3β1 and αvβ3 pathways. Cell proliferation, migration, apoptosis, or tube formation ability were detected, and the expression of integrin-associated regulatory proteins was detected. The anti-tumor activity of T7 peptide, meloxicam, and their combination were evaluated in HCC tumor models established in mice. T7 peptide suppressed the proliferation, migration, tube formation, and promoted the apoptosis of endothelial cells under both normoxic and hypoxic conditions via integrin α3β1 and αvβ3 pathways. Meloxicam enhanced the activity of T7 peptide under hypoxic condition. T7 peptide partly inhibited COX-2 expression via integrin α3β1 not αvβ3-dependent pathways under hypoxic condition. T7 peptide regulated apoptosis associated protein through MAPK-dependent and -independent pathways under hypoxic condition. The MAPK pathway was activated by the COX-2/PGE2 axis under hypoxic condition. The combination of T7 and meloxicam showed a stronger anti-tumor effect against HCC tumors in mice. The data highlight that meloxicam enhanced the antiangiogenic activity of T7 peptide *in vitro* and *in vivo*.

## Introduction

Angiogenesis is the critical event in the process of tumor development, in which tumors develop additional new blood vessels to acquire sufficient nutrients and oxygen (Mashreghi et al., 2018; Tu et al., 2021). Hypoxia and nutrient deprivation initiate an ‘angiogenic switch’ to promote tumor growth once a tumor diameter exceeds a few millimeters (Zhou et al., 2020). The angiogenic switch triggers tumor cells to release a series of cytokines and growth factors, which stimulate the proliferation and sprouting of endothelial cells accompanied with matrix metalloproteinases (MMPs)-mediated degradation of vascular basement membranes (VBMs), resulting in the exposure of some fragments of VBMs, which can function as endogenous angiogenic inhibitors (Fernando et al., 2008; Morse et al., 2019). Effective endogenous antiangiogenic molecules have demonstrated a huge therapeutic potential for cancer treatment (Munir et al., 2020). Several endogenous angiogenic inhibitors have been identified through the degradation of VBMs mediated by MMPs (Fields, 2019; Ricard-Blum & Vallet, 2019). One such molecule is tumstatin, a 244-amino acid poly-peptide fragment from the noncollagenous 1 domain of the α3-chain of type IV collagen (Ricard-Blum & Vallet, 2019).

Although tumstatin displays potent antitumor activities (Hamano et al., 2003; Boosani et al., 2007), its clinical use is still limited due to its high molecular weight, low solubility, and immunogenicity (Boosani et al., 2010; Esipov et al., 2012). The T7 peptide, located in the N-terminal half of tumstatin and restricted to amino acids 74–98, has a molecular weight of only 3.02 kDa, and displays the similar inhibitory activity to tumstatin on the proliferation of endothelial cells (Sudhakar & Boosani, 2008; Wang et al., 2013). However, the molecular mechanisms and its restricted anti-angiogenic activity under hypoxic conditions are still obscure (Liu et al., 2019; Najafi et al., 2020).

Tissues of solid tumors including HCC contain hypoxic microenvironments, which can be further increased by antiangiogenic drugs (Liang et al., 2013; Muz et al., 2019). Tumor hypoxic microenvironment also hinders antiangiogenic drugs to exert their therapeutic effects (Sormendi & Wielockx, 2018). Cyclooxygenase-2 (COX-2) is a major actor in hypoxic endothelial cells (Zhao et al., 2012). Prostaglandin E2 (PGE2), the predominant product of COX-2, executes diverse biological effects of COX-2 primarily through its binding to a family of receptors (Dong et al., 2018; Tong et al., 2018). Therefore, the COX-2/PGE2 axis may be involved in T7-mediated anti-angiogenic and anti-tumor activities; but the mechanisms for its role remain obscure and need further investigation.

## Materials and methods

### Reagents, antibodies, and kits

The T7 peptide (T7) (H-Thr-Met-Pro-Phe-Leu-Phe-Cys-Asn-Val-Asn-Asp-Val-Cys-Asn-Phe-Ala-Ser-Arg-Asn-Asp-Tyr-Ser-Tyr-Trp-Leu-OH) was synthesized by GL Biochem Ltd. (Shanghai, China) and dissolved in 5% acetic acid. Antibodies (Abs) against p44/42 MAPK (referred to as MAPK), phosphorylated (p)-p44/42MAPK (referred to as p-MAPK), myeloid cell leukemia-1 (Mcl-1), Bcl-2, Bax, survivin, proliferating cell nuclear antigen (PCNA), CD34, and GAPDH were purchased from Cell Signaling Technology (Danvers, MA). Abs against integrin αIIb and COX-2 were from Santa Cruz Biotechnology (Santa Cruz, TX). Abs against integrins α3 and β3 were purchased from ImmunoWay Biotechnology Company (Newark, DE). Abs against CD3, integrins αv and β1 were purchased from Abcam (Cambridge, UK). PGE2 and meloxicam were purchased from Merck Millipore (Merck Millipore, Darmstadt, Germany). Human VEGF ELISA Kit was purchased from Abcam (Cambridge, UK). Integrin β3 siRNA (sc-29375), integrin α3 siRNA (sc-35684), COX-2 siRNA (sc-29729), negative control siRNA (NC siRNA, sc-37007), and siRNA transfection reagent (sc-29528) were purchased from Santa Cruz Biotechnology (Santa Cruz, TX).

### Cell culture

Human umbilical vein endothelial cells (HUVECs) and human pulmonary microvascular endothelial cells (HPMECs) were purchased from Typical Animal Reserve Center of China (Shanghai, China) and ScienCell (Carlsbad, CA), respectively. Both HUVECs and HPMECs were cultured in endothelial cell medium (ECM, ScienCell, Carlsbad, CA) and cells harvested at passages 3–10 were used in assays. Human hepatocellular carcinoma (HCC) Hep3B cells were obtained from ATCC and cultured in DMEM supplemented with 10% FBS, 100 U/mL penicillin, and 100 mg/mL streptomycin. Human lung carcinoma cells (A549) were obtained from the China Centre for Type Culture Collection (CCTCC, Wuhan, China) and cultured in RPMI-1640 supplemented with 10% FBS, 100 U/mL penicillin, and 100 mg/mL streptomycin. Human erythroleukemia TF-1 cells were purchased from ATCC and cultured in RPMI-1640 supplemented with 2 ng/mL rhGM-CSF and 10% FBS. Human glioblastoma U87-MG cells were from ATCC (Molsheim, France) and cultured in DMEM supplemented with 10% FBS, 100 U/mL penicillin, and 100 mg/mL streptomycin. All the above cells were incubated at 37 °C under 95% air and 5% CO_2_. A hypoxic condition was created in a hypoxia chamber (Billups-Rothenberg, Inc., San Diego, CA) equilibrated with certified gas containing 1% O_2_, 5% CO_2_, and 94% N_2_.

### Transfection of siRNAs

Integrin α3 siRNA, integrin β3 siRNA, and negative control siRNA were transfected into HUVECs and HPVECs by using the siRNA transfection reagent according to the manufacturer’s instructions.

### Cell viability assay, cell migration assay, ELISA, and immunoblotting analysis

The methods have been previously described (Dong et al., 2014, 2018).

### Tube formation assay

The endothelial cell tube formation assay was performed as previously described (Wagenblast et al., 2015). Briefly, Matrigel^TM^ Matrix (BD Biosciences, San Jose, CA) was pre-coated on a 96-well plate and incubated for 1 h at 37 °C. HUVECs (5 × 10^3^ cells/well) or HPMECs (5 × 10^3^ cells/well) were added on top of the Matrigel^TM^ Matrix and incubated for 8 h. Images were randomly taken from three different fields (×100 magnification) of each well and the number of capillary structures were counted.

### Apoptosis assay

Cells were incubated with 5 µL of Annexin V and 5 µL of propidium iodide (PI) for 15 min at room temperature in the dark, according to the manufacturer’s instruction (BD Biosciences, San Jose, CA), and then subjected to flow cytometry to measure the apoptosis rate (%).

### Xenograft animal model

All surgical procedures and care given to the animals were in accordance with institutional guidelines as described in our published studies (Xiu et al., 2013; Dong et al., 2018). Briefly, subcutaneous Hep3B tumors were established in nude mice, and tumor dimensions and volumes were determined every two days. When tumors reached approximately 100 mm^3^, the mice were randomly divided into four groups, which received administration of vehicle (control), meloxicam, T7, and meloxicam + T7. Meloxicam was orally given at a dose of 20 mg/kg/d, T7 was intraperitoneally injected at a dose of 4.4 mg/kg/d. The mice were closely monitored and tumors were measured and harvested at the end of experiments.

### Assessment of tumor vascular density and Ki-67 proliferation index

The micro-vessel density (MVD) and Ki-67 proliferation index were determined as previously described (Dong et al., 2018). Briefly, 4 μm paraffin tumor sections were stained with anti-CD34 and anti-Ki67 Abs. Stained vessels of five randomly chosen fields served as the number of MVD. The Ki-67-positive cells were counted in five randomly selected fields under microscopy. The Ki-67 proliferation index was calculated according to the following formula: number of Ki-67 positive cells/total cell count × 100%.

### Assessment of cell apoptosis *in situ*

TUNEL staining of tumor sections was performed using an *in situ* apoptosis detection kit (Roche, Shanghai, China) and the method was performed as previously described (Dong et al., 2018).

### Patients and clinical samples

The clinical study had been approved by the ethical committees of the People’s Hospital of Guangxi Zhuang Autonomous Region (Nanning, China) and Shandong Cancer Hospital and Institute, Shandong First Medical University, and Shandong Academy of Medical Sciences (Jinan, China). A total of 30 human HCC tissue samples were included in the study. The expression of CD34 (a marker of endothelial cells) in primary tumors, portal vein cancerous thrombus, and bile duct cancerous thrombus was detected by immunohistochemistry to observe the morphological functions of endothelial cells. Consecutive serial sections (4 μm) stained for CD34 and CD3 (a specific marker of T lymphocytes) were used to analyze the relationship between endothelial cells and tumor immune activity.

### Statistical analysis

Statistical analysis was performed with SPSS 21.0 software (SPSS Inc., Chicago, IL). All tests were two-tailed, and *p* < .05 was considered statistically significant.

## Results

### T7 suppresses the proliferation of endothelial cells through integrin α3β1 and αvβ3 dependent pathways

**Figure 1.**
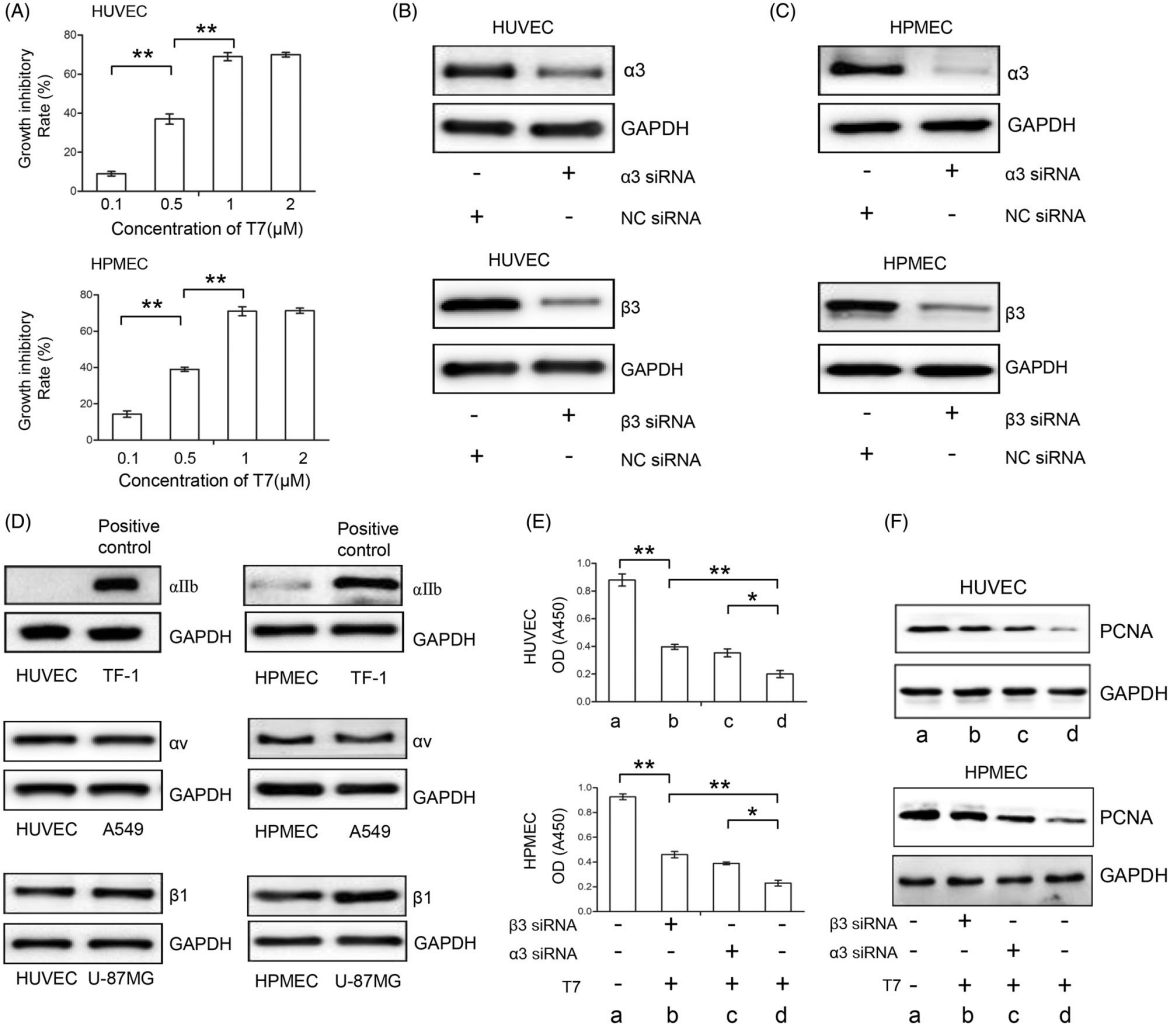
T7 suppresses proliferation of endothelial cells through integrin α3β1 and αvβ3-dependent pathways. (A) HUVECs and HPMECs were incubated in endothelial cell medium containing T7 at indicated concentrations and the growth inhibitory rate was calculated. (B, C) HUVECs and HPMECs were transfected with α3 siRNA or β3 siRNA, or negative control siRNA (NC siRNA) and 48 h later integrin α3 and β3 expressions were detected by Western blot. (D) Western blot analysis for integrin subunit αIIb in HUVECs and HPMECs (TF-1 cells were used as a positive control, the top panel); Western blot analysis for integrin subunit αv in HUVECs and HPMECs (A549 cells were used as a positive control, the middle panel); Western blot analysis for integrin subunit β1 in HUVECs and HPMECs (U-87MG cells were used as a positive control, the bottom panel). (E) HUVECs and HPMECs with or without the treatment of α3 siRNA, β3 siRNA, or T7 were cultured for 72 h and the cell viability (OD value) was measured. (F) The above cells in (D) were subjected to Western blot to detect expression of PCNA. The band density was measured and normalized to that of GAPDH. Data represent three independent experiments. (a) No specific treatment; (b) treated with T7 and β3 siRNA; (c) treated with T7 and α3 siRNA; (d) treated with T7. *A significant (*p*<.05) difference; **A highly significant (*p*<.001) difference.

As shown in Figure 1(A), T7 significantly inhibited the proliferation of HUVECs and HUMECs in a concentration-dependent manner and at a concentration of 1 μmol/L, the inhibitory activity of T7 reached the peak. Integrin α3 or β3 subunits with other subunits form functional heterodimer α3β1, αvβ3, and αIIbβ3 (Figure S1) (Wong et al., 2020), and integrin α3β1 and αvβ3 are shown to be involved in the regulation of tumor angiogenesis and potential ligands of fragments generated from tumstatin (Ricard-Blum & Vallet, 2019). Transfections of siRNA targeting integrin α3 or β3 efficiently reduced their expression (Figure 1(B,C)), and integrin subunit αIIb was absent (Figure 1(D)), suggesting that downregulation of integrin α3 or β3 subunit can abrogate the function of integrin heterodimers αvβ3 or α3β1 in human endothelial cells. Transfection of siRNA targeting integrin α3 or β3 subunit partially abolished the anti-proliferative effects of T7 in HUVECs and HUMECs (Figure 1(E)). Western blotting analysis of PCNA, a cell proliferation marker, confirmed the effects of T7 on cell proliferation (Figure 1(F)). These results indicated that T7 inhibits the proliferation of endothelial cells through integrin α3β1 and αvβ3-dependent pathways.

### T7 suppresses the migration and tube formation of endothelial cells by interacting with integrin α3β1 and αvβ3

**Figure 2.**
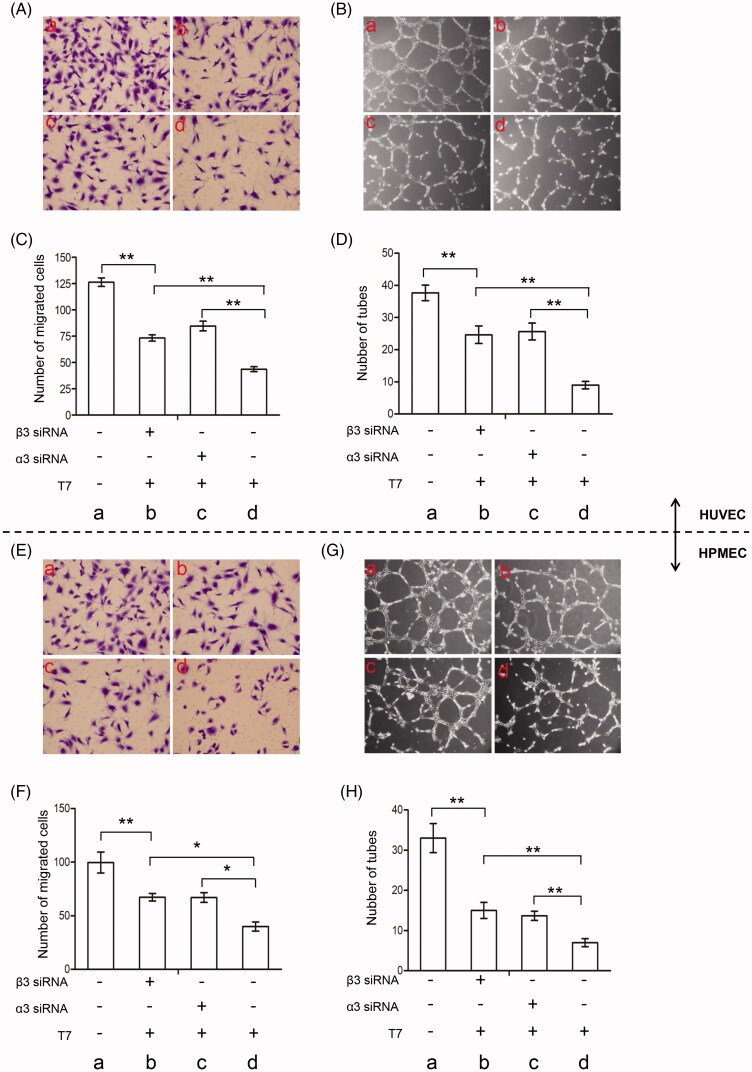
T7 suppresses migration and tube formation of endothelial cells by binding with integrin α3β1 and αvβ3. (A, B) HUVECs were treated with or without T7, α3 siRNA, or β3 siRNA and subjected to cell migration assay. The migrated cells were photographed and quantified (×400 magnification). Data represent three independent experiments. **A highly significant (*p*<.01) difference. (C, D) HUVECs were treated with or without T7, α3 siRNA, or β3 siRNA and subjected to tube formation assay. The tubes were photographed and quantified (×100 magnification). Data represent three independent experiments. (E, F) HPMECs were treated with or without T7, α3 siRNA, or β3 siRNA and subjected to cell migration assay. The migrated cells were photographed and quantified (×400 magnification). Data represent three independent experiments. (G, H) HPMECs were treated with or without T7, α3 siRNA, or β3 siRNA and subjected to tube formation assay. The tubes were photographed and quantified (×100 magnification). Data represent three independent experiments. *A significant (*p*<.05) difference; **A highly significant (*p*<.01) difference.

Compared with controls, T7 significantly inhibited the migration of HUVECs (126.3 ± 4.06 vs. 43.67 ± 2.33, *p* < .01), and knockdown of integrin subunit α3 (84.63 ± 4.63 vs. 43.67 ± 2.33), or β3 (73.33 ± 2.96 vs. 43.67 ± 2.33) partly abrogated this effect of T7 (Figure 2(A,B)). Compared with controls, T7 also significantly inhibited the tube formation of HUVECs on the Matrigel matrix (37.67 ± 2.40 vs. 9.00 ± 1.16, *p* < .01), and knockdown of integrin subunit α3 (25.67 ± 2.60 vs. 9.00 ± 1.16), or β3 (24.67 ± 2.78 vs. 9.00 ± 1.16) partly abolished this effect of T7 peptide (Figure 2(C,D)).

Similarly, compared with controls, T7 also significantly inhibited the migration of HPMECs (99.67 ± 5.61 vs. 40.00 ± 2.52, *p* < .01), and knockdown of integrin subunit α3 (67.00 ± 2.65 vs. 40.00 ± 2.52) or β3 (67.33 ± 2.03 vs. 40.00 ± 2.52) partly abrogated this effect of T7 (Figure 2(E,F)). In addition, compared with controls, T7 significantly inhibited the tube formation of HPMECs on the Matrigel matrix (33.00 ± 2.08 vs. 7.00 ± 0.58, *p* < .01), and knockdown of integrin subunit α3 (13.60 ± 0.67 vs. 7.00 ± 0.58, *p* < .01) or β3 (15.00 ± 1.16 vs. 7.00 ± 0.58, *p* < .01) partly abrogated this effect of T7 (Figure 2(G,H)). The results indicated that T7 suppresses the migration and tube formation of endothelial cells by interacting with integrin α3β1 and αvβ3.

### T7 regulates the apoptosis of endothelial cells through integrin α3β1 and αvβ3-dependent pathways

**Figure 3.**
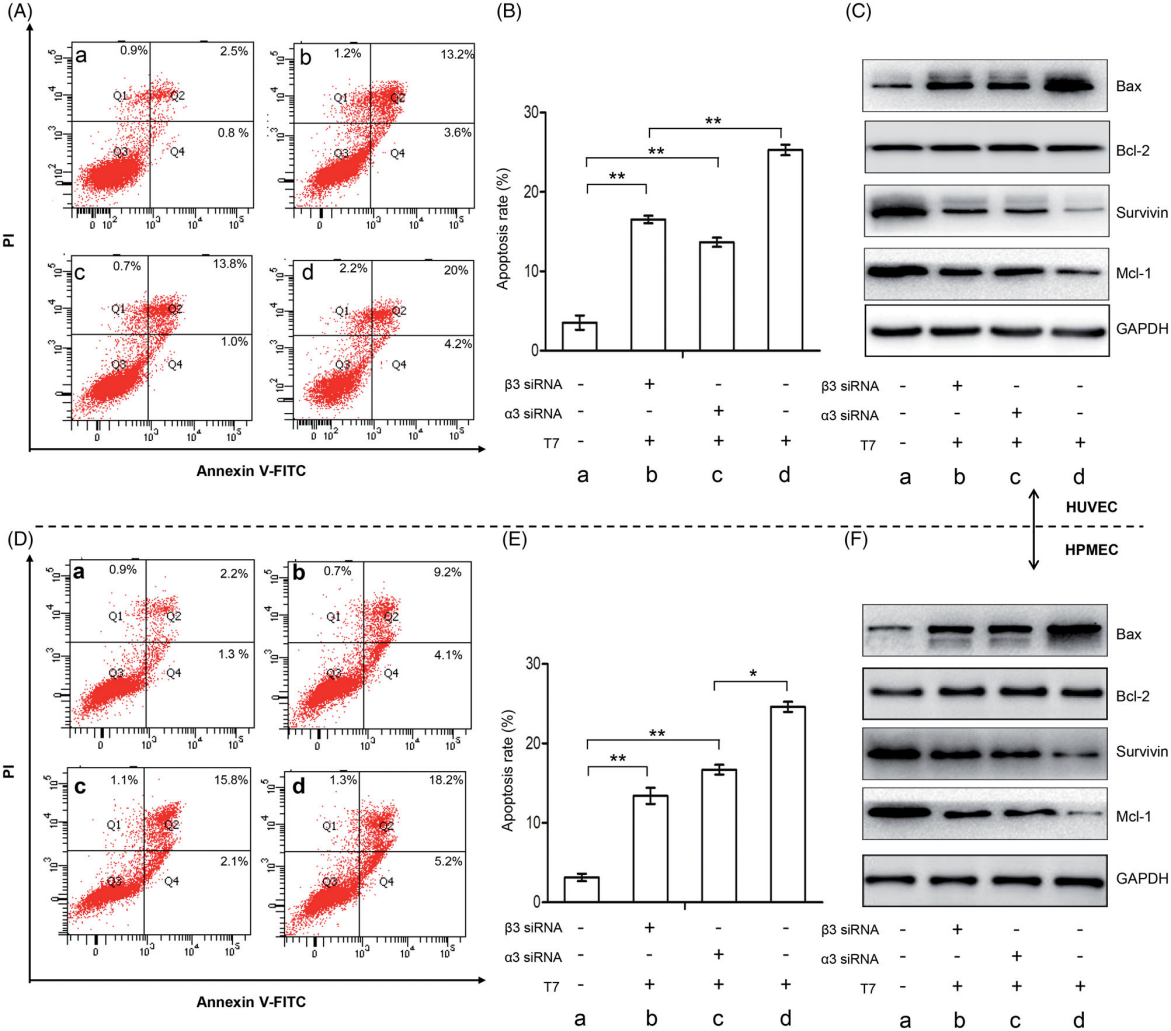
T7 regulates apoptosis of endothelial cells through integrin α3β1 and αvβ3-dependent pathways. (A, B) HUVECs treated with or without T7, α3 siRNA, or β3 siRNA, were subjected to flow cytometry to measure the apoptosis rate (%). (C) HUVECs were treated with or without T7, α3 siRNA, or β3 siRNA, and the apoptosis related proteins Bax, Bcl-2, survivin, and Mcl-1 were detected by Western blot analysis. GAPDH served as an internal control. (D, E) HPMECs treated with or without T7, α3 siRNA, or β3 siRNA, were subjected to flow cytometry to measure the apoptosis rate (%). (F) HPMECs were treated with or without T7, α3 siRNA, or β3 siRNA, and the apoptosis related protein Bax, Bcl-2, survivin, and Mcl-1 were detected by Western blot analysis. GAPDH served as an internal control. *A significant (*p*<.05) difference; **A highly significant (*p*<.001) difference.

T7 significantly increased the apoptosis rate of HUVECs compared with controls (25.30 ± 0.67% vs. 3.53 ± 0.90%, *p* < .01), and knockdown of integrin subunit α3 (13.67 ± 0.58% vs. 25.30 ± 0.67%, *p* < .01), or β3 (16.53 ± 0.48% vs. 25.30 ± 0.67%, *p* < .01) could partly abolish this effect of T7 (Figure 3(A,B)). In the mechanism exploration, we found that T7 significantly increased the expression of pro-apoptosis protein Bax, and decreased the expression of anti-apoptosis proteins Mcl-1 and survivin in HUVECs cells, but the expression of anti-apoptosis protein Bcl-2 remained unchanged upon T7 treatment in HUVECs (Figure 3(C)). Knockdown of integrin subunit α3 or β3 could partly abolish this effect of T7 in HUVEC cells as well.

Similarly, T7 also significantly increased the apoptosis rate of HPMECs compared with controls (24.60 ± 0.64% vs. 3.13 ± 0.47%, *p* < .01), and knockdown of integrin subunit α3 (16.70 ± 0.61% vs. 24.60 ± 0.64%, *p* < .01), or β3 (13.40 ± 1.01% vs. 24.60 ± 0.64%, *p* < .01) could partly abolish this effect of T7 (Figure 3(D,E)). In HPMECs, the alteration in expression of mitochondrial-associated apoptotic proteins (Bax, Mcl-1, and Bcl-2) and apoptosis inhibitor (survivin) upon T7 exposure and gene knock-down of integrin subunit α3 or β3 showed the similar trend (Figure 3(F)) to those in HUVEC cells. The present studies indicated that T7 can induce the apoptosis of endothelial cells via integrin α3β1 and αvβ3-dependent pathways.

### The COX-2/PGE2 contributes to hypoxia-mediated apoptosis resistance of endothelial cells to T7

**Figure 4.**
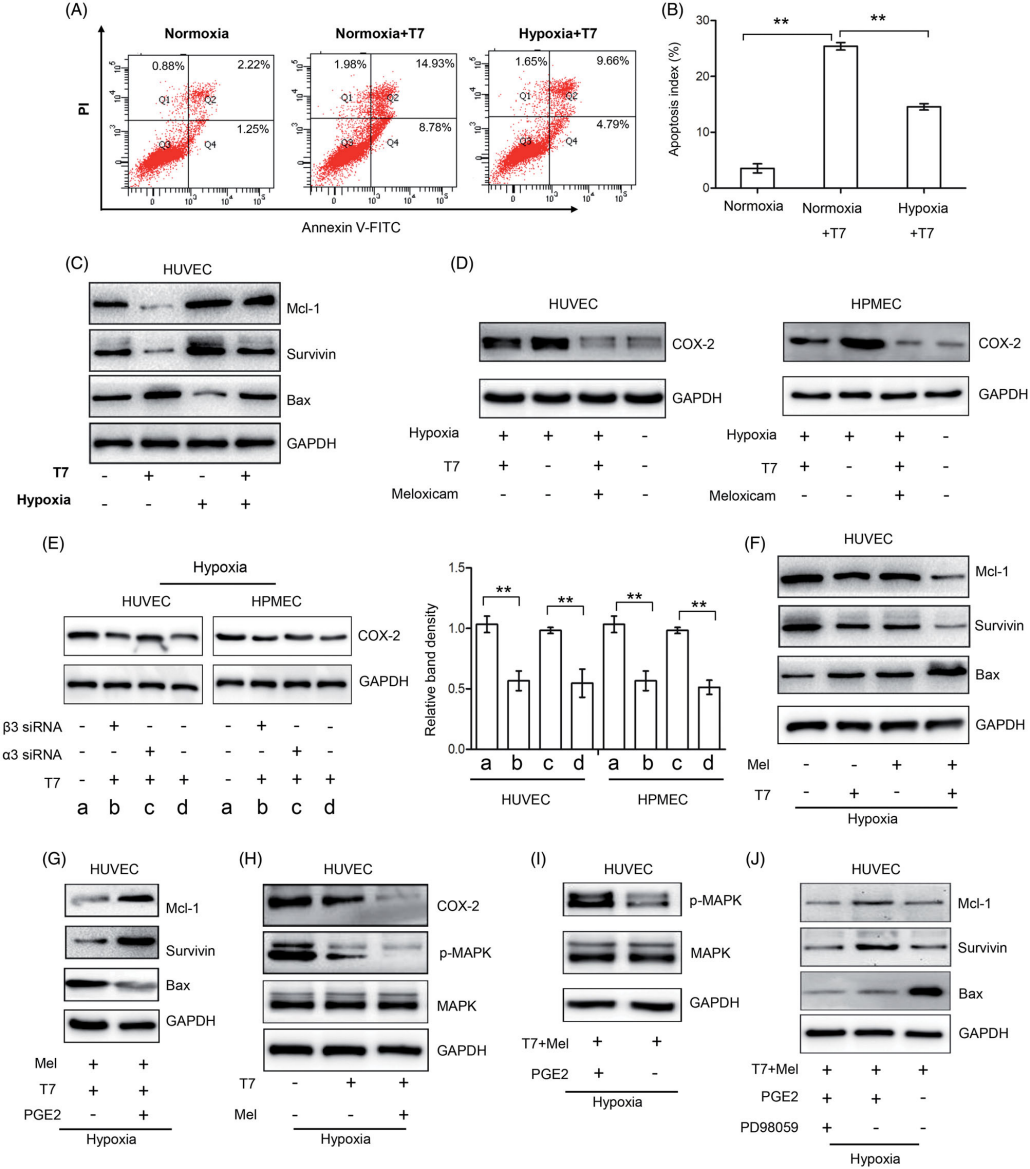
Combined targeting of the COX-2/PGE2 axis enhanced the pro-apoptotic activity of T7 in endothelial cells under hypoxia. (A, B) HUVECs treated with or without T7 under normoxic or hypoxic conditions and were subjected to flow cytometry to detect cell apoptosis (%). (C) HUVECs were treated with or without T7 or meloxicam (80 μM) under hypoxia, and apoptosis related proteins Bax, survivin, and Mcl-1 were detected by Western blot analysis. (D) HUVECs or HPMECs were treated with or without T7 under normoxic or hypoxic conditions, and COX-2 expression was detected by Western blot analysis. (E) HUVECs or HPMECs were treated with or without T7, α3 siRNA, or β3 siRNA under hypoxic conditions, and COX-2 expression was detected by Western blot analysis. (F) HUVECs were treated with or without T7, α3 siRNA, or β3 siRNA under hypoxic conditions, and COX-2 expression was detected by Western blot analysis. (G) HUVECs were treated with or without T7, α3 siRNA, or β3 siRNA under hypoxic conditions, and COX-2 expression was detected by Western blot analysis. (G–J) HUVECs were treated with or without T7, meloxicam (80 μM), PGE2 (3 μM), PD98059 (50 μM), or a combination under hypoxic conditions, and expressions of Mcl-1, Bax, survivin, COX-2, MAPK, and p-MAPK were detected by Western blot analysis. GAPDH served as an internal control. The band density was measured and normalized to that of GAPDH. **A highly significant (*p*<.001).

Tumor hypoxic microenvironment activates a series of signaling pathways (Wu et al., 2015; Dong et al., 2018), which enhance the angiogenesis of malignant tumors (Wu et al., 2015; Dong et al., 2018). Here, we also found that compared with T7 treatment under normoxic conditions, hypoxia effectively reduced the pro-apoptosis activity of T7 on HUVECs, compared with those treated with T7 under normoxia (14.55 ± 0.57% vs. 25.40 ± 0.66%, *p* < .01) (Figure 4(A,B)). T7 could significantly downregulate the expression of Mcl-1 and survivin and upregulate the expression of Bax in HUVECs under normoxia, which hypoxia reduced these effects of T7 (Figure 4(C)). T7 peptide did not show any effect on the regulation of Bcl-2 under either normoxia or hypoxia in either HUVECs or HPMECs (Figure S2). It has been reported that the COX-2/PGE2 axis participates in the biological response of endothelial cells to hypoxia and regulates apoptosis-associated proteins (Zhao et al., 2012). Here, we also demonstrated that the expression of COX-2 was significantly increased in HUVECs and HPMECs exposed to hypoxia, T7 peptide could partly block the upregulation of COX-2 induced by hypoxia, and the combination of meloxicam or celecoxib (both are COX-2/PGE2 specific inhibitors) and T7 almost thoroughly abolished the increased COX-2 expression induced by hypoxia in HUVECs and HPMECs (Figure 4(D)). The ratio of T7/meloxicam is optimized based on our experiments and the coefficient of drug interaction (CDI) was utilized to display the effects of interaction between meloxicam and T7 (Figure S3). However, T7 failed to suppress hypoxia-induced upregulation of COX-2 in HUVECs and HPMECs when integrin α3 but not β3 was depleted by specific siRNA (Figure 4(E)). Overall, the results indicated that T7 can inhibit hypoxia-induced COX-2 expression only through integrin α3β1-dependent pathway.

In agreement with previous studies (Zhao et al., 2012; Wang et al., 2019), the combination of T7 and meloxicam resulted in further upregulation of Bax and downregulation of Mcl-1 and survivin compared to T7 alone (Figure 4(F)). However, PGE2 could reverse the regulatory effects of the combination of T7 and meloxicam on the expression of apoptosis-associated proteins in HUVECs under hypoxia (Figure 4(G)). The above results indicate that T7 peptide may induce apoptosis of HUVECs through the COX-2/PGE2-dependent pathway.

The activation of the MAPK signal pathway is an important event in the apoptosis resistance in malignant tumors under hypoxia (Liu et al., 2010; Dong et al., 2018). Here, we showed that T7 could only partly inhibit the phosphorylation of MAPK, and the combination of T7 and meloxicam almost completely inhibited the phosphorylation of MAPK (Figure 4(H)), but this effect could be reversed by the addition of PGE2 (Figure 4(I)). However, when PD98059, an MAPK-specific inhibitor, was added, PGE2 failed to the suppressive effects on the expression of Mcl-1 and survivin by the combination of T7 and meloxicam (Figure 4(J)), although PD98059 had no effect on the expression of Bax regulated by T7 and meloxicam (Figure 4(J)). The results suggest that COX-2/PGE2 can stimulate the activation of MAPK, leading to the alteration of Mcl-1 and survivin expression under hypoxia, and the expression of Bax regulated by the COX-2/PGE2 axis was in a MAPK-independent pathway in endothelial cells.

### The COX-2/PGE2 axis activates the MAPK pathway involved in the regulation of proliferation, migration, and tube formation of endothelial cells under hypoxia

**Figure 5.**
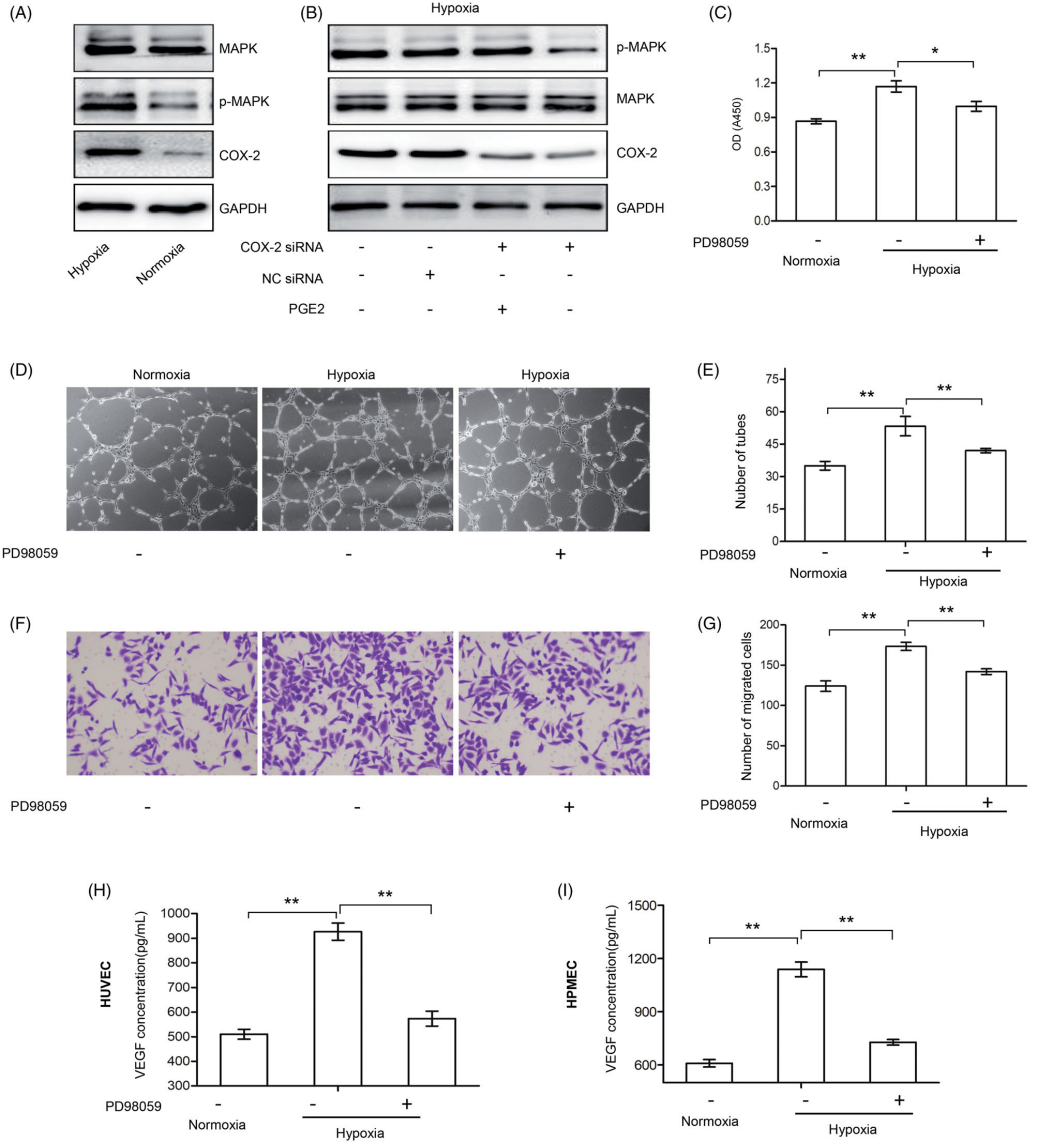
Hypoxia promoted the activation of MAPK via the COX-2/PGE2 axis in endothelial cells under hypoxic conditions. (A, B) HUVECs were treated with or without COX-2 siRNA, NC siRNA, or PGE2 (3 μM) under normoxic or hypoxic conditions, and expressions of MAPK, p-MAPK, and COX-2 were detected by Western blot analysis. GAPDH served as an internal control. (C) HUVECs were treated with or without PD98059 (50 μM) under normoxic or hypoxic conditions for 24 hours and the cell viability assay was detected. (D, E) HUVECs were treated with or without PD98059 (50 μM) under normoxic or hypoxic conditions and were subjected to tube formation assay. The tubes were photographed and quantified (×100 magnification). Data represent three independent experiments. (F–G) HUVECs were treated with or without PD98059 (50 μM) under normoxic or hypoxic conditions and were subjected to cell migration assay. The migrated cells were photographed and quantified (×100 magnification). Data represent three independent experiments. (H, I) HUVECs (H) or HPMECs (I) were treated with or without PD98059 (50 μM) under normoxic or hypoxic conditions for 24 hours. The VEGF concentrations in the supernatant were measured by ELISA assay. *A significant (*p*<.05) difference; **A highly significant (*p* < .01) difference.

The expression of COX-2 and phosphorylated MAPK was increased in HUVECs under hypoxia (Figure 5(A)). The knockdown of COX-2 reduced the activation of MAPK, which was reversed by the addition of PGE2 (Figure 5(B)). The results further demonstrated that the COX-2/PGE2 axis regulates MAPK activation under hypoxic conditions.

The proliferation migration, and tube formation of HUVECs were augmented by hypoxia, but this effect was blocked by the addition of PD98059 (Figure 5(C–G)). VEGF is the critical regulator of endothelial cell activity (Apte et al., 2019). We showed here that hypoxia mediated upregulation of VEGF was blocked by the addition the PD98059 in HUVECs and HPMECs (Figure 5(H,I)).

### Endothelial cells have an important role in HCC development and HCC is a suitable biological model for endothelial activity research

**Figure 6.**
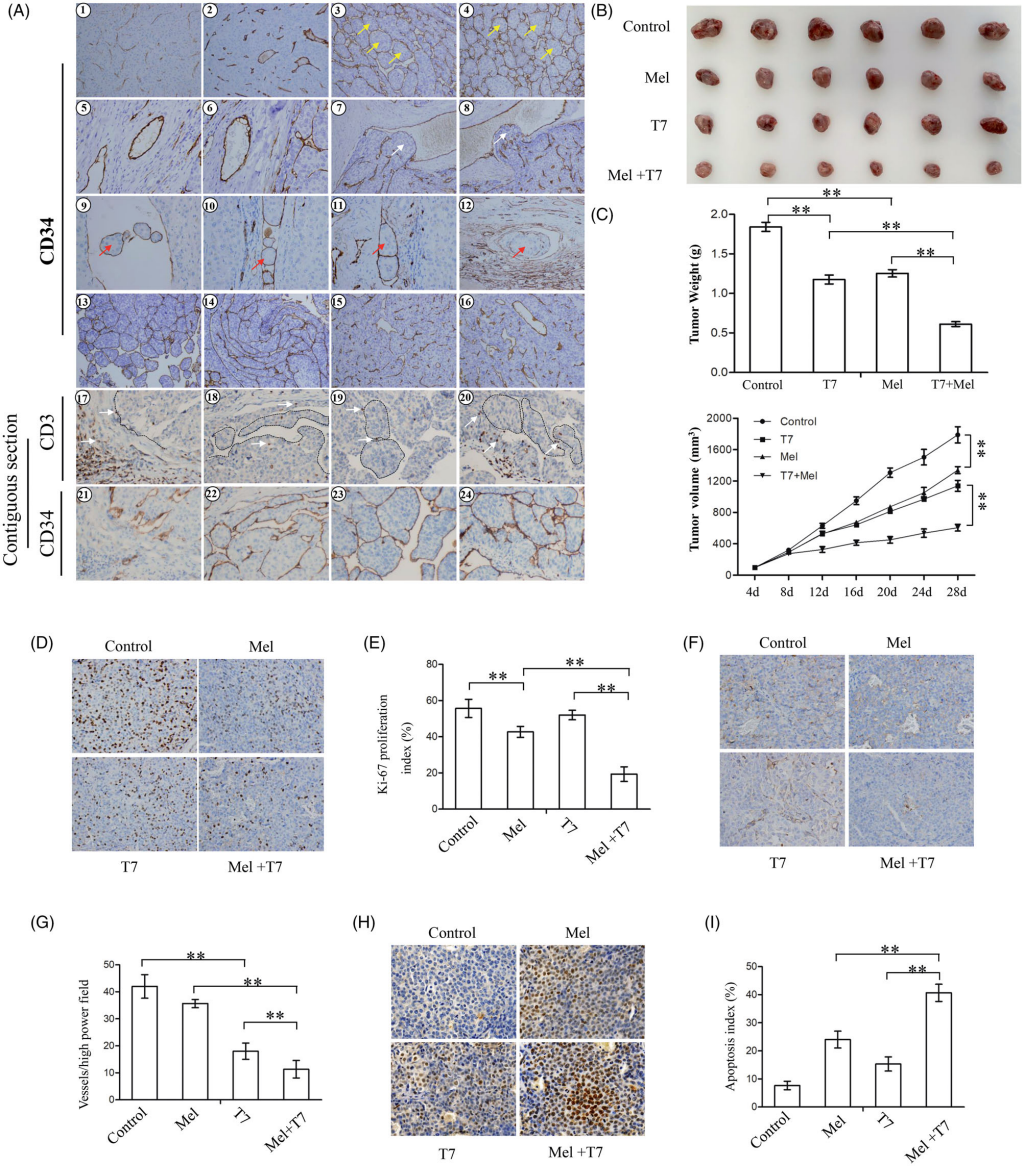
Detection of the various functions of endothelial cells in HCC patients and the synergistic effects of COX-2-specific inhibitors and T7 in inhibiting the growth of xenografts of HCC cells in nude mice. (A) Representative HCC cases were analyzed by IHC staining for CD34 and CD3. 1–4: HCC samples for CD34 staining; 5, 6: cancer or para-carcinoma tissues for CD34, yellow ‘→’ indicates spherical vesicles formed by endothelial cells; 7, 8: cancer tissues for CD34, white ‘→’ indicates tumor cell mass which is invading into vascular wall into blood stream; 9–12: para-carcinoma tissues for CD34, red ‘→’ indicates spherical cancer nests circulating in the blood stream; 13, 14: portal vein cancerous thrombus for CD34 staining; 15, 16: bile duct cancerous thrombus for CD34 staining; 17–20: immunohistochemistry of paraffin sections of HCC (17, 18) and portal vein cancerous thrombus (19, 20) for CD3 (a pan‐T lymphocyte marker) staining, white ‘→’ indicates T lymphocyte; 21–24: immunohistochemistry of paraffin sections of HCC (21, 22) and portal vein cancerous thrombus (23, 24) for CD34 staining, which are contiguous sections corresponding to 17–20 (magnification 1–4: ×200; 5, 6: ×400; 7, 8: ×200; 9–12: ×200; 13–16: ×200; 17–24: ×400). (B, C) Hep3B tumors were established in mice, which were treated meloxicam, T7, or a combination as described in ‘Materials and Methods’ section. All the tumors obtained from nude mice at the end of the experiments are imaged. The tumors were excised, and the tumor size/weight were measured at the end of the experiments. (D–I) Representative tumor sections prepared from nude mice in the control group, meloxicam group, T7 group, and T7 + meloxicam group were prepared. Tumor sections were stained with an anti-Ki-67 antibody to detect the proliferating cells (D), an anti-CD34 antibody to detect the microvessels (F), and TUNEL agent to detect the apoptotic cells (H). Ki67 positive cells were counted to calculate the Ki-67 proliferation index (E), tumor microvessels in sections were counted in randomly chosen fields to record microvessel density (G), and TUNEL-positive cells were counted to calculate the apoptosis index (I). Data represent three independent experiments. **a highly significant (*p*<.001) difference.

Endothelial cells are the key cells in the process of angiogenesis (Petrou, 2018), and HCC is a classical hypervascular tumor. We thus examined the functions of endothelial cells in HCC tissues by using immunohistochemistry. With the immunostaining of CD34, endothelial cells display two different morphological structures, namely common capillary vessels and spherical structures. Based on the endothelial structure, HCC can be classified into type 1 and type 2, respectively. In type 1 HCC, endothelial cells formed tube-like vessels, namely common capillary vessels (Figure 6(A): 1, 2). In type 2 HCC, endothelial cells envelop HCC cell masses into spherical structures (Figure 6(A): 3, 4). Endothelial cells abscise from vessel walls and form spherical vesicles, which may act as a trap to capture more endothelial cells and change the hemodynamics in tumor microenvironment (Figure 6(A): 5, 6). In metastasis of malignant tumors, high shear stress from the blood stream can suppress cell proliferation and migration, promote apoptosis, and break tumor cells into debris presenting a negative regulatory factor for cancer metastasis (Lee et al., 2017; Ji et al., 2019). Here, we found that endothelial cells could protect HCC cells from the high shear stress of the blood stream by encapsulating tumor cell masses in the process of invading into vascular walls (Figure 6(A): 7, 8).

Endothelial cells could also envelop HCC cells into spherical cancer nests and migrate into vessels (Figure 6(A): 9–12). The spherical cancer nests were observed in portal vein thrombus of type 2 HCC tissues (Figure 6(A): 13, 14), and no spherical cancer nests were observed portal vein thrombus of type 1 HCC patients (Figure S4). In bile duct cancerous thrombus, rare spherical cancer nests were observed in both type 1 (Figure 6(A): 15) and type 2 HCC tissues (Figure 6(A): 16). Endothelial cells can metastasize along with tumor cells in portal vein cancerous thrombus (Figure 6(A): 13, 14) and bile duct cancerous thrombus (Figure 6(A): 15, 16). Compared with primary tumors, HCC cells in the cancerous thrombus in bile or portal vein cancerous showed a higher proliferation index and almost non-existent tumor stroma in addition to endothelial cells (Figure S5). The results suggest that endothelial-mediated metastasis may be a key step to help tumor cells escape from the stroma (such as fibroblasts and collagen) and acquire a higher proliferation ability.

In order to detect the possible immunoprotective effects of endothelial cells, immunohistochemistry of CD34 (a marker of endothelial cells) and CD3 (a specific marker of T lymphocytes) analysis was carried out. Immunohistochemistry of consecutive paraffin sections of HCC tissues containing portal vein cancerous thrombi by using Abs again CD34 and CD3 showed that endothelial cells or endothelial vessels act as a potential barrier to protect T lymphocytes to contact with tumor cells (Figure 6(A): 17–24). Interestingly, a small number of T lymphocytes were also found in bile duct cancerous thrombi, indicating that T lymphocytes may migrate along with HCC cells (Figure S6).

### Meloxicam enhances the antitumor activity of T7 against HCC xenografts in mice

Subcutaneous Hep3B tumors were established in mice, which were assigned to different treatments. The untreated control tumors grew remarkably quickly, reaching 1842 ± 138 mm^3^ after 24 days treatment. In contrast, in the meloxicam group, tumors reached only 1254 ± 114 mm^3^, which was significantly smaller than control tumors (*p* < .05). In the T7 group, tumors were 1176 ± 127 mm^3^ in volume and highly significantly smaller than control tumors (*p* < .01). The combination of meloxicam and T7 resulted in an even smaller tumors with a volume of only 611 ± 75 mm^3^, significantly smaller than those treated with meloxicam or T7 alone (both *p* < .05) (Figure 6(B,C)). The data of tumor volumes were supported by those of tumor weights (Figure 6(C)).

A further analysis of tumors harvested from the mice showed a significant reduction of MVD and Ki-67 proliferation index in the combination group compared with T7 or meloxicam alone (Figure 6(D–G)), and a significant increase of the apoptosis index in the combination group (Figure 6(H,I)).

## Discussion

The present study found that T7 regulated endothelial cell proliferation, migration, tube formation, and apoptosis through integrin α3β1 and αvβ3-dependent pathways. Hypoxia significantly restricted the activity of T7. Under hypoxic conditions, T7 could mediated endothelial cell apoptosis through integrin α3β1 and αvβ3-dependent pathways and exclusively regulate apoptosis-related proteins Bax, Mcl-1, and survivin through the integrin α3β1 pathway. T7 could only partly suppress the activity of the COX-2/PGE2 axis via the integrin α3β1 pathway. The COX-2/PGE2 axis mediates the activation of the MAPK pathway followed by the upregulation of VEGF (Figure S7). The combination of T7 and the COX-2 inhibitor meloxicam enhanced the effect of T7 in inhibiting the growth of HCC tumors in nude mice (Borza et al., 2006).

Integrins are bidirectional hubs transmitting signals between cells and their microenvironment in malignant tumors (Sokeland & Schumacher, 2019; Wang et al., 2019). In the process of anti-angiogenesis therapy of malignant tumors, tumor hypoxic environments are increased and the persistent hypoxic challenge subject cells to a series of functions to antagonize the pro-apoptotic effects of chemotherapeutic drugs (Najafi et al., 2020; Phung et al., 2020). COX-2 is a key apoptosis-resistant factor, and it is significantly increased in endothelial cells under hypoxic conditions (Zhong et al., 2015; Tong et al., 2018). When a hypoxic microenvironment occurs, different integrin dimers may transmit specific biological signals and regulate the expression of survival-associated proteins (Cao et al., 2019). In this study, we found that upregulated COX-2 expression induced by hypoxia inhibited the pro-apoptotic activity of T7 in endothelial cells. T7 could only partly suppress the expression of COX-2 by binding the integrin α3β1 dimer rather than the αvβ1 dimer in hypoxic endothelial cells. With the assistance of the COX-2/PGE2 specific inhibitor, T7 almost abolished COX-2 expression in hypoxic endothelial cells and significantly promoted cell apoptosis. The results indicated that under hypoxic conditions, the integrin α3β1 dimer may be the key candidate in regulating hypoxia-associated apoptosis protein expression. In consideration of the formation of hypoxic environments, the combination of meloxicam and T7 in the therapy of HCC subcutaneous xenografts in nude mice is necessary. The present study showed that the combined therapy of meloxicam and T7 had a stronger suppression against HCC tumors via the regulation of cell proliferation, MVD, and cell apoptosis.

Anti-angiogenic therapy, has become one of the most important means in treating malignant tumors by blocking the supply of oxygen and nutrients of tumors (Folkman, 1971; Grizzi et al., 2020). In the present study, we found that endothelial cells could not only form vessels to supply oxygen and nutrients for HCC but could also envelop tumor cells to protect them from blood flow shear stress and immune attack (Figure 6(A)). Therefore, we propose that tumor vasculature associated therapy should be more accurately defined as ‘anti-endothelial cell therapy’ rather than ‘anti-angiogenesis therapy’. When endothelial cells are inhibited, tumor clusters lose the protective layer, resulting in more tumor cells being directly attacked by immune cells and blood flow shear stress (Figure 6(A)). In the present study, we found that the tumor mass in blood vessels was completely wrapped by endothelial cells, which may protect tumor cells from immune attack and blood flow impingement (Figure 6: 9–12).

Immuno-checkpoint blocking therapy targeting the PD-1 or PD-L1 has become a new approach to the treatment of malignant tumors, but drug resistance has reduced its effectiveness (Wang & Wu, 2020). Although the possible factors have been proposed for explaining the resistance to PD-1 or PD-L1 monoclonal Abs, the role of endothelial cells is ignored (Wang & Wu, 2020). Our previous studies showed that T7 inhibited proliferation and promoted apoptosis of endothelial cells, indicating that the addition of T7 may enhance the effectiveness of PD-1 or PD-L1 monoclonal Abs (Wang et al., 2015; Wang & Wu, 2020). Meanwhile, another observation worthy further investigation is that immune cells need to cross endothelial cell layer to attack HCC cells (Figure 6(A): 17–24). Endothelial cells may be first attacked by anti-endothelial cell drugs (such as T7), resulting in cell apoptosis and endothelial wall broken, and immune cells can then filtrate into tumor clusters and execute their function. In our previous study, we also found that T7 could also partly reduce HCC cell viability and induce cell cycle arrest, and T7 peptide maybe an important drug candidates in the treatment of malignancies (Liu et al., 2019).

Therefore, we designed the present study to investigate the mechanisms accounting for anti-angiogenesis therapy or anti-endothelial cell therapy of T7, and its combination with the COX-2/PGE2 specific inhibitor. In conclusion, combined therapies targeting the COX-2/PGE2 axis may enhance the anti-angiogenic and antitumor activities of T7.

## Supplementary Material

Supplemental Material

## Data Availability

The data that support the findings of this study are available from the corresponding author upon reasonable request.
