# Supplementary material for: Targeting of the COX-2/PGE2 axis enhances the antitumor activity of T7 peptide in vitro and in vivo
Source: Drug Deliv. 2021 Apr 30;28(1):844–55. doi: 10.1080/10717544.2021.1914776 (PMC8812588; doi:10.1080/10717544.2021.1914776)
Supplement: Supplemental Material [file IDRD_A_1914776_SM2471.docx]

**Supplementary MATERIALS**

**Supplementary FIGURE 1 Representation of the integrin family.** In humans, the integrin family contains 24 heterodimers and integrin subunit α3 only exists in heterodimer α3β1 and integrin subunit β3 only exists in heterodimers αvβ3, αIIbβ3.

**Supplementary FIGURE 2 Detection of Bcl-2 expression in endothelial cells.**

HUVECs or HPMECs were treated with or without T7 under normoxic or hypoxic conditions. The total cell protein was extracted, and Bcl-2 expression was detected by Western blot analysis. GAPDH served as an internal control.

Supplementary FIGURE 3 Effect of meloxicam (Mel) and T7 peptide individual and in combination on the viability of HUVECs. A, Cell viability was assessed by the CCK-8 cell viability assay. HUVECs cells were exposed to Mel and T7 peptide alone or incombination for 24 h under hypoxic conditions. Date are expressed as the percentage of control cells and are the means±SD of three separate experiments. ^*^*P*＜0.05; ^**^*P*＜0.01 vs. Mel alone, ^#^ *P*＜0.05; **^# #^** *P*＜0.01 vs. T7 alone. B, The coefficient of drug interaction (CDI) of the combination of Mel and T7 in HUVECs.

**Supplementary FIGURE 4** Using IHC staining for CD34 in human portal vein cancerous thrombus of Type 1 HCC patients (200×)

A-D, Typical pictures in which endothelial cells combine into common capillary vessels in human portal vein cancerous thrombus of HCC.

**Supplementary FIGURE 5 Detection of cell proliferation of primary tumors, portal vein cancerous thrombus, and bile duct cancerous thrombus by Ki-67 staining in Type 1 HCC and Type 2 HCC patients.**

Using IHC staining for Ki-67 for primary tumors, portal vein cancerous thrombus and bile duct cancerous thrombus of HCC patients (400×). A, Type 1 HCC, typical pictures in which Ki-67 positive cells showed a different density in primary tumors, portal vein cancerous thrombus, and bile duct cancerous thrombus of HCC patients. B, Type 2 HCC, typical pictures in which Ki-67 positive cells showed a different density in primary tumors, portal vein cancerous thrombus and bile duct cancerous thrombus of HCC patients.

**Supplementary FIGURE 6 Immunohistochemistry of paraffin sections of bile duct cancerous thrombus for CD3 and CD34 staining (400×).** Using IHC staining for CD34 and CD3 (a pan‐T lymphocyte marker) stain，white *“→” indicates* T lymphocyte*,* in human contiguous sections of bile duct cancerous thrombus of HCC patients (400×). A-B, Type 1 HCC. C-D, Type 2 HCC.

**Supplementary FIGURE 7 Proposed mechanisms by which T7 executes its anti-endothelial cell activity under normoxic and hypoxic conditions with the cooperation of COX-2/PGE2 specific inhibitors.**

T7 suppresses proliferation, migration, and tube formation and promotes apoptosis of endothelial cells through both integrin α3β1 and αvβ3 dependent pathways under normoxic conditions. Hypoxia restricts the activity of T7. Hypoxia upregulates COX-2 expression followed by the phosphorylated activation of MAPK, which blocks the anti-endothelial cell activity of T7 via upregulating the anti-apoptosis protein Mcl-1/survivin. The COX-2/PGE2 axis also blocks T7-mediated upregulation of pro-apoptosis protein Bax through the phosphorylated MAPK independent pathway. The phosphorylated MAPK promotes proliferation, migration, and tube formation of endothelial cells via the upregulation of VEGF. Under hypoxic conditions, T7 could partly inhibit COX-2 expression via integrin α3β1 rather than αvβ3 dependent pathway. With the assistance of COX-2/PGE2 specific inhibitors (meloxicam or celecoxib), T7 could still retain its anti-endothelial cell activity under hypoxic conditions. “→” indicates positive regulation or activation; “⊥”, negative regulation or blockade; COX-2, cyclooxygenase-2; PGE2, prostaglandin E2; Mel, meloxicam; Cel, celecoxib.
